# Nutrition and Eye Health

**DOI:** 10.3390/nu11092123

**Published:** 2019-09-06

**Authors:** John G. Lawrenson, Laura E. Downie

**Affiliations:** 1 Centre for Applied Vision Research, School of Health Sciences, City University of London, London EC1V 0HB, UK; 2 Department of Optometry and Vision Sciences, The University of Melbourne, Parkville, Victoria 3010, Australia

Diet is a key lifestyle factor that can have long-term effects on ocular health. This Special Issue of Nutrients entitled ‘Nutrition and Eye Health’ contains 12 articles, including reviews and primary research studies, that report on a diverse range of topics relating to the role of nutrition in maintaining eye health, and the potential use of nutritional interventions for preventing or treating ocular disease. Collectively, these papers span a spectrum of ocular conditions, including corneal angiogenesis [1], cataract [2,3,4], diabetic retinopathy [5], age-related macular degeneration (AMD) [6,7], and experimental models of retinal disease [8,9,10]. In addition, clinically focussed papers report on the validation of a novel food frequency questionnaire for assessing long-chain omega-3 fatty acid intake in eye care practice [11], and evidence relating to the applicability of saffron for treating ocular disease [12].

Globally, approximately 250 million people suffer from varying degrees of vision loss [13]. Leading causes include several eye conditions considered in this Special Issue, such as cataract, AMD, glaucoma, and diabetic retinopathy. These conditions disproportionately affect older adults, and with an ageing population the number of affected individuals is predicted to increase exponentially [13]. Whilst the aetiology of age-related eye disease is complex and multifactorial, oxidative stress has been implicated as a common causative mechanism. The eye is particularly susceptible to oxidative stress as a result of its high oxygen consumption, high concentration of polyunsaturated fatty acids and cumulative exposure to high-energy visible light. This combination of factors leads to the generation of reactive oxygen species that can trigger oxidative damage to ocular tissues. Consequently, there has been significant research interest in the role of dietary antioxidants and the potential therapeutic benefits of antioxidant vitamin and mineral supplements as a simple and cost-effective strategy for disease prevention and/or control [14,15,16,17].

AMD is characterised by degenerative changes within the macula, the central area of the retina that is responsible for high-resolution vision, in people aged 55 years or older. AMD is a leading cause of severe vision impairment in European-derived populations. In the UK, the disease is responsible for over 50% of certifiable vision loss [18]. Although epidemiological studies have provided reasonably consistent evidence that diet is an important modifiable risk factor for AMD [19], concerns have been raised about the validity of findings from non-interventional studies due to the potential influence of confounding factors. For example, people with a particular dietary pattern may differ in other ways (e.g., the amount of exercise they undertake, their daily level of light exposure) and it is not typically possible to control for these differences [20].

In terms of primary research studies, the highest quality evidence to evaluate the efficacy and safety of therapeutic interventions derives from randomised controlled trials (RCTs). In RCTs, participants are randomly allocated to receive either the intervention or a comparator (typically placebo or no intervention), which minimises the potential for bias in the intervention assignment [21]. There is evidence from RCTs that prophylactic antioxidant vitamin or mineral supplementation does not prevent the development of AMD [22]. Five large RCTs have compared supplements containing vitamin E, beta-carotene, vitamin C, or antioxidant vitamin combinations with placebo in people from the general population. These trials randomised more than 75,000 people and followed their clinical outcomes between 4 to 10 years. People taking these supplements were found to have a similar risk of developing AMD to those not taking the supplements [22].

Other RCTs have investigated whether high-dose antioxidant vitamin and mineral supplements can slow the progression of AMD [23]. Most of these trials recruited small numbers of participants and were of relatively short duration, ranging from 9 months to 6 years. However, one large, multi-centre RCT conducted in the USA, the Age-Related Eye Disease Study (AREDS), randomised 3640 individuals with AMD to take supplement formulations containing combinations of vitamin C, E, beta-carotene, zinc, and copper, or a placebo, each day. A major conclusion from the AREDS was that daily, long-term supplementation with vitamin C (500 mg), vitamin E (400 international units (IU)), beta-carotene (15 mg), zinc (80 mg, as zinc oxide), and copper (2 mg, as cupric oxide) reduced the relative risk of progression to late-stage AMD from 28% (observed with placebo) to 20% at 5 years, in people with at least intermediate AMD. This means that for people with intermediate AMD, who are at the highest risk of progression to late AMD, 80 fewer cases would progress for every 1000 people taking the supplement. However, safety concerns were raised regarding high-dose supplementation of the carotenoid used in the original AREDS supplement, beta-carotene, in people who smoke [24]. In a follow-up study by the AREDS investigators, AREDS2, current smokers or those who had ceased smoking for less than 12 months before enrolment were not eligible to receive beta-carotene supplementation [25]. The primary analysis in AREDS2 demonstrated that adding lutein and zeaxanthin and/or omega-3 fatty acids to the AREDS formula was not associated with a significant reduction in the risk of progression to late-stage AMD compared with the original supplement. Lutein and zeaxanthin are carotenoids that are major components of macula pigment. They are proposed to have a protective role in the retina through their antioxidant properties and ability to act as a filter for blue light [26]. Exploratory analyses from AREDS2 suggested that lutein and zeaxanthin may be of value for reducing AMD progression when given without beta-carotene, but that more research was required to test this hypothesis [27].

Cataract is defined as any visible opacity within the otherwise clear crystalline lens of the eye. Cataract can be further classified as cortical, nuclear, or posterior sub-capsular, depending on the anatomical location of the opacity. Globally, over 60 million people are visually impaired due to cataract, however cataract-associated blindness shows significant geographical variation, accounting for less than 22% of blindness in high-income countries compared to more than 44% in South East Asia [13]. Age is the most significant risk factor for cataractogenesis. As the lens ages, conformational changes to lens proteins occur with subsequent aggregation, leading to a progressive loss of transparency and associated vision loss [28]. Oxidation reactions within the lens are thought to be a key factor in this process and there has been a significant amount of research on the role of antioxidant nutrients for preventing or slowing the progression of cataract [29]. Observational data suggest that the risk of cataracts can be reduced by a diet that contains optimal levels of vitamins C and E, the carotenoids lutein and zeaxanthin, and the daily use of multivitamin supplements [29]. However, RCTs that have compared antioxidant vitamin supplements (beta-carotene, vitamins C and E) to an inactive placebo or no supplement have been unable to detect any effect on the incidence or progression of cataract [30]. The lack of efficacy in these relatively short-term trials could suggest that a longer-term intake or a particular combination of antioxidants is required. A study of baseline factors that predicted cataract in the AREDS cohort found that the use of multivitamins supplements reduced the risk of developing nuclear cataracts over an approximate 10 year follow-up period [31].

In conclusion, age-related eye diseases, including cataract and AMD, are of global public health concern. Acquired vision loss associated with these conditions can be devastating to the individual through its detrimental impact on quality of life, and also impart substantial societal burden. Although the pathogenesis of these conditions is not fully understood, there is increasing evidence that their impact can, to some extent, be mitigated by targeting modifiable risk factors. Since diet and nutrition have been linked with the most common diseases affecting the elderly, dietary modification and nutritional supplementation for the prevention and treatment of these diseases has attracted a considerable amount of scientific attention. As evidenced by the quality and diversity of the contributions in this Special Issue, the role of nutrition in eye health remains a highly topical area, with scope for future research to enhance our understanding of the role of nutritional strategies for optimising eye health.

## References

[1] Estrella-Mendoza M.F., Jimenez-Gomez F., Lopez-Ornelas A., Perez-Gutierrez R.M., Flores-Estrada J. (2019). Cucurbita argyrosperma Seed Extracts Attenuate Angiogenesis in a Corneal Chemical Burn Model. Nutrients.

[2] Braakhuis A.J., Donaldson C.I., Lim J.C., Donaldson P.J. (2019). Nutritional Strategies to Prevent Lens Cataract: Current Status and Future Strategies. Nutrients.

[3] Lim V., Schneider E., Wu H., Pang I.H. (2018). Cataract preventive role of isolated phytoconstituents: Findings from a decade of research. Nutrients.

[4] Zych M., Wojnar W., Dudek S., Kaczmarczyk-Sedlak I. (2019). Rosmarinic and Sinapic Acids May Increase the Content of Reduced Glutathione in the Lenses of Estrogen-Deficient Rats. Nutrients.

[5] Rossino M.G., Casini G. (2019). Nutraceuticals for the treatment of diabetic retinopathy. Nutrients.

[6] Lawrenson J.G., Evans J.R., Downie L.E. (2019). A Critical Appraisal of National and International Clinical Practice Guidelines Reporting Nutritional Recommendations for Age-Related Macular Degeneration: Are Recommendations Evidence-Based?. Nutrients.

[7] Rinninella E., Mele M.C., Merendino N., Cintoni M., Anselmi G., Caporossi A., Gasbarrini A., Minnella A.M. (2018). The role of diet, micronutrients and the gut microbiota in age-related macular degeneration: New perspectives from the gut(-)retina axis. Nutrients.

[8] Morita Y., Miwa Y., Jounai K., Fujiwara D., Kurihara T., Kanauchi O. (2018). Lactobacillus paracasei KW3110 Prevents Blue Light-Induced Inflammation and Degeneration in the Retina. Nutrients.

[9] Kang M.K., Lee E.J., Kim Y.H., Kim D.Y., Oh H., Kim S.I., Kang Y.H. (2018). Chrysin ameliorates malfunction of retinoid visual cycle through blocking activation of AGE-RAGE-ER stress in glucose-stimulated retinal pigment epithelial cells and diabetic eyes. Nutrients.

[10] Yu M., Yan W., Beight C. (2018). Lutein and Zeaxanthin Isomers Protect against light-induced retinopathy via decreasing oxidative and endoplasmic reticulum stress in BALB/cJ Mice. Nutrients.

[11] Zhang A.C., Downie L.E. (2019). Preliminary Validation of a Food Frequency Questionnaire to Assess Long-Chain Omega-3 Fatty Acid Intake in Eye Care Practice. Nutrients.

[12] Heitmar R., Brown J., Kyrou I. (2019). Saffron (*Crocus sativus* L.) in Ocular Diseases: A Narrative Review of the Existing Evidence from Clinical Studies. Nutrients.

[13] Flaxman S.R., Bourne R.R.A., Resnikoff S., Ackland P., Braithwaite T., Cicinelli M.V., Das A., Jonas J.B., Keeffe J., Kempen J.H. (2017). Global causes of blindness and distance vision impairment 1990–2020: A systematic review and meta-analysis. Lancet Glob. Health.

[14] Weikel K.A., Chiu C.J., Taylor A. (2012). Nutritional modulation of age-related macular degeneration. Mol. Asp. Med..

[15] Sideri O., Tsaousis K.T., Li H.J., Viskadouraki M., Tsinopoulos I.T. (2019). The potential role of nutrition on lens pathology: A systematic review and meta-analysis. Surv. Ophthalmol..

[16] Loskutova E., O’Brien C., Loskutov I., Loughman J. (2019). Nutritional supplementation in the treatment of glaucoma: A systematic review. Surv. Ophthalmol..

[17] Li C., Miao X., Li F., Wang S., Liu Q., Wang Y., Sun J. (2017). Oxidative Stress-Related Mechanisms and Antioxidant Therapy in Diabetic Retinopathy. Oxid. Med. Cell. Longev..

[18] Quartilho A., Simkiss P., Zekite A., Xing W., Wormald R., Bunce C. (2016). Leading causes of certifiable visual loss in England and Wales during the year ending 31 March 2013. Eye.

[19] Chiu C.J., Taylor A. (2007). Nutritional antioxidants and age-related cataract and maculopathy. Exp. Eye Res..

[20] Downie L.E., Keller P.R. (2014). Nutrition and age-related macular degeneration: Research evidence in practice. Optom. Vis. Sci..

[21] Ioannidis J.P. (2013). Implausible results in human nutrition research. BMJ.

[22] Evans J.R., Lawrenson J.G. (2017). Antioxidant vitamin and mineral supplements for preventing age-related macular degeneration. Cochrane Database Syst. Rev..

[23] Evans J.R., Lawrenson J.G. (2017). Antioxidant vitamin and mineral supplements for slowing the progression of age-related macular degeneration. Cochrane Database Syst. Rev..

[24] Tanvetyanon T., Bepler G. (2008). Beta-carotene in multivitamins and the possible risk of lung cancer among smokers versus former smokers: A meta-analysis and evaluation of national brands. Cancer.

[25] Group A.R., Chew E.Y., Clemons T., SanGiovanni J.P., Danis R., Domalpally A., McBee W., Sperduto R., Ferris F.L. (2012). The Age-Related Eye Disease Study 2 (AREDS2): Study design and baseline characteristics (AREDS2 report number 1). Ophthalmology.

[26] Arunkumar R., Calvo C.M., Conrady C.D., Bernstein P.S. (2018). What do we know about the macular pigment in AMD: The past, the present, and the future. Eye.

[27] Chew E.Y., Clemons T.E., Sangiovanni J.P., Danis R.P., Ferris F.L., Elman M.J., Antoszyk A.N., Ruby A.J., Orth D., Fish G.E. (2014). Secondary analyses of the effects of lutein/zeaxanthin on age-related macular degeneration progression: AREDS2 report No. 3. JAMA Ophthalmol..

[28] Michael R., Bron A.J. (2011). The ageing lens and cataract: A model of normal and pathological ageing. Trans. R. Soc. B Biol. Sci..

[29] Weikel K.A., Garber C., Baburins A., Taylor A. (2014). Nutritional modulation of cataract. Nutr. Rev..

[30] Mathew M.C., Ervin A.M., Tao J., Davis R.M. (2012). Antioxidant vitamin supplementation for preventing and slowing the progression of age-related cataract. Cochrane Database Syst. Rev..

[31] Chang J.R., Koo E., Agron E., Hallak J., Clemons T., Azar D., Sperduto R.D., Ferris F.L., Chew E.Y. (2011). Age-Related Eye Disease Study Group. Risk factors associated with incident cataracts and cataract surgery in the Age-related Eye Disease Study (AREDS): AREDS report number 32. Ophthalmology.

